# Development of a One-Step Multiplex PCR Assay for Differential Detection of Four species (*Enterobacter cloacae*, *Enterobacter hormaechei*, *Enterobacter roggenkampii*, and *Enterobacter kobei*) Belonging to *Enterobacter cloacae* Complex With Clinical Significance

**DOI:** 10.3389/fcimb.2021.677089

**Published:** 2021-05-18

**Authors:** Yang Ji, Peihong Wang, Tingting Xu, Yanzi Zhou, Rongchang Chen, Huaiqiu Zhu, Kai Zhou

**Affiliations:** ^1^ Shenzhen Institute of Respiratory Diseases, Second Clinical Medical College, Jinan University (Shenzhen People’s Hospital); The First Affiliated Hospital, Southern University of Science and Technology (Shenzhen People’s Hospital), Shenzhen, China; ^2^ State Key Laboratory for Turbulence and Complex Systems and Department of Biomedical Engineering, College of Engineering, Peking University, Beijing, China; ^3^ Center for Quantitative Biology, Peking University, Beijing, China; ^4^ Collaborative Innovation Center for Diagnosis and Treatment of Infectious Diseases, State Key Laboratory for Diagnosis and Treatment of Infectious Diseases, The First Affiliated Hospital, College of Medicine, Zhejiang University, Hangzhou, China

**Keywords:** multiplex PCR, Enterobacter cloacae complex, Enterobacter cloacae, Enterobacter hormaechei, Enterobacter roggenkampii and Enterobacter kobei

## Abstract

*Enterobacter cloacae* complex (ECC) is composed of multiple species and the taxonomic status is consecutively updated. In last decades ECC is frequently associated with multidrug resistance and become an important nosocomial pathogen. Currently, rapid and accurate identification of ECC to the species level remains a technical challenge, thus impedes our understanding of the population at the species level. Here, we aimed to develop a simple, reliable, and economical method to distinguish four epidemiologically prevalent species of ECC with clinical significance, i.e., *E. cloacae*, *E. hormaechei*, *E. roggenkampii*, and *E. kobei*. A total of 977 ECC genomes were retrieved from the GenBank, and unique gene for each species was obtained by core-genome comparisons. Four pairs of species-specific primers were designed based on the unique genes. A total of 231 ECC clinical strains were typed both by *hsp60* typing and by species-specific PCRs. The specificity and sensitivity of the four species-specific PCRs ranged between 96.56% and 100% and between 76.47% and 100%, respectively. The PCR for *E. cloacae* showed the highest specificity and sensitivity. A one-step multiplex PCR was subsequently established by combining the species-specific primers. Additional 53 *hsp60*-typed ECC and 20 non-ECC isolates belonging to six species obtained from samples of patients, sewage water and feces of feeding animals were tested by the multiplex PCR. The identification results of both techniques were concordant. The multiplex PCR established in this study provides an accurate, expeditious, and cost-effective way for routine diagnosis and molecular surveillance of ECC strains at species level.

## Introduction

*Enterobacter* spp. are natural commensals of the animal and human gut microbiota (Sanders and Sanders, 1997; Mesa et al., 2006). Over the past decades, some members of *Enterobacter* spp. have become important nosocomial pathogens thus are classified as one of the “ESKAPE” bugs (*Enterococcus faecium*, *Staphylococcus aureus*, *Klebsiella pneumoniae*, *Acinetobacter baumannii*, *Pseudomonas aeruginosa*, and *Enterobacter* species) (Rice, 2008). Among *Enterobacter* spp., the *E. cloacae* complex (ECC) is of major importance, accounting for 65%–75% of infections in clinical settings (Sanders and Sanders, 1997). The ECC is frequently associated with a multidrug resistance (MDR) phenotype, mainly due to the ability to easily acquire numerous genetic mobile elements carrying resistance genes. Moreover, the chromosome of ECC intrinsically encodes *ampC* genes, resulting in a constitutive production of the AmpC β-lactamase and is thus intrinsically resistant to ampicillin, amoxicillin, amoxicillin/clavulanate, first-generation cephalosporins, and cefoxitin (Mezzatesta et al., 2012). Available epidemiological data show that the ECC has become the third major drug-resistant *Enterobacteriaceae* species involved in nosocomial infections after *Escherichia coli* and *K. pneumoniae* (Zhou et al., 2018). Hence, it is important to control the dissemination of MDR-ECC for public health.

According to physiology and biochemistry phenotypes, as well as genotypes, more than 20 species and clades (without taxonomy terms) are classified into ECC till now (Sutton et al., 2018), and the taxonomic status is consecutively updated. Of note, increasing studies show that the clinical importance and drug resistance of different ECC members is significantly different. A number of epidemiological studies reveal that *E. hormaechei* is the most prevalent species in the clinical setting (Morand et al., 2009; Chavda et al., 2016; Moradigaravand et al., 2016), and this species is commonly considered a causative pathogen responsible for various infections (Davin-Regli et al., 1997; Paauw et al., 2009; Mezzatesta et al., 2012). Another important member of ECC is *E. kobei*. Clinical strains of *E. kobei* have been isolated from various clinical samples, including blood, sputum, urine, bronchoalveolar lavage, abscesses, and especially intra-abdominal samples (Zhou et al., 2017; Davin-Regli et al., 2019). A recent study showed that *E. hormaechei* and *E. kobei* were the predominant ESBL-positive species accounting for more than 70% of community-acquired ECC strains collected across China (Zhou et al., 2018). *E. cloacae* is very often isolated in samples of clinical origin and is found frequently during systematic sampling of neonates who have been colonized early. The species is involved in 10% of postsurgical peritonitis cases, 5% of nosocomial sepsis and pneumonia cases, and 4% of urinary tract infections (Davin-Regli et al., 2019). A large genomic study by collecting 316 MDR-ECC isolates from bloodstream infections between 2001 and 2011 across hospitals in the UK and Ireland shows that *E. hormaechei*, *E. kobei*, and *E. cloacae* account for more than 80% of the studied population, suggesting the epidemiological and clinical significance for the three species (Moradigaravand et al., 2016). *E. roggenkampii* is a recently described species based on a computational analysis of sequenced *Enterobacter* genomes, and can cause multi-site infection (e.g. blood, urine, feces, and body fluids). The species has widely distributed worldwide, including Australia, the United States, Germany, and China, etc. (Xu et al., 2021). Collectively, the data highly support the necessity of studying ECC on species level.

Currently, the accurate identification of ECC species and subspecies remains a challenge. In clinical laboratories, the routine identification of ECC is mainly dependent on phenotypic characteristics by using commercialized systems, like Vitek-II (bioMérieux) and the Matrix-assisted laser desorption/ionization-time-of-flight (MALDI-TOF) mass spectrometry (MS) technology. Although these systems can identify the ECC, they fail to differentiate the species within the group (Davin-Regli et al., 2019). Molecular methods are more suitable for precisely identification of the ECC on species level, and currently *hsp60* typing is the most widely used method for this purpose. By sequencing and phylogenetically analyzing the *hsp60* gene, Hoffman and Roggenkamp grouped the ECC into 12 genetic clusters (I–XII) and one unstable sequence cluster (XIII) (Hoffmann and Roggenkamp, 2003). Additionally, the technique of microarray comparative genomic hybridization (microarray-CGH) (Paauw et al., 2008), multi-locus sequence analysis (MLSA) (Singh et al., 2018), and whole-genome sequencing (Chavda et al., 2016) show the power on the species-level identification of ECC. However, these methods are time-consuming, expensive, and therefore difficult to implement routinely. Recently, a qPCR-based method targeting the *hsp60* gene was developed and showed high accuracy to detect *E. hormaechei* (Ohad et al., 2014). The limited detection spectrum cannot fulfill the needs of clinical diagnosis yet.

Given that current methods have significant disadvantages, there is a need to develop an efficient and accurate method to identify the ECC population on the species level for routine diagnosis and research. The primary goal of our study is to setup and optimize a one-step multiplex PCR assay capable of differentiating the major ECC species (i.e. *E. cloacae*, *E. hormaechei*, *E. roggenkampii*, and *E. kobei*) with clinical significance. Our optimized multiplex PCR assay is able to provide a reliable and rapid detection method for laboratory diagnosis to employ in the clinical setting.

## Materials and Methods

### Bacterial Collection

A total of 284 ECC and 20 non-ECC strains were used in this study, including 294 clinical isolates, eight environmental isolates recovered from hospital sewage water and two livestock-associated isolates recovered from feces of farmed animals. Species identification was performed by using VITEK MS (bioMerieux, Marcy-l’Étoile, French). The bacteria were cultured in Luria-Bertani (LB) liquid (Tryptone 10 g, NaCl 10 g, Yeast extract 5 g, H_2_O 1L, pH 7.0) or agar medium. The bacterial genomic DNA were extracted using the conventional boiling lysis method as previously described (Chen et al., 2011).

### Bioinformatic Analyses and Design of Species-Specific Primers

A total of 977 ECC genomes were retrieved from the NCBI RefSeq database as of June 2018. The average nucleotide identity (ANI) value between each genome and the type strain of each species/subspecies (i.e. *E. cloacae* ssp. *cloacae* ATCC 13047, *E. cloacae* ssp. *dissolvens* SDM, *E. hormaechei* ssp. *hormaechei* ATCC 49162, *E. hormaechei* ssp. *oharae* DSM 16687, *E. hormaechei* ssp. *steigerwaltii* DSM 16691, *E. hormaechei* subsp*. Xiangfangensis*, *E. roggenkampii* DSM 16690, and *E. kobei* DSM 13645) was calculated by using RYANI (Pritchard et al., 2016) to accurately identify the species of each genome, with a cut-off of 95%. The genomes of *E. hormaechei*, *E. kobei*, *E. roggenkampii*, and *E. cloacae* identified were re-annotated using Prokka (Seemann, 2014). The annotated genomic sequences were used as the input to Roary (Page et al., 2015) to calculate the core and accessory genes of each species. The identity threshold was set at 90% without split paralogs. The unique genes of each species were defined as the core genes present in more than 95% genomes of the species but less than 5% genomes of the other species. The pair-wise blast was further performed for the unique genes to exclude sequences with high homology (30% of amino acid sequence identity and 10% of sequence coverage). The resulting unique genes were selected as the candidates of species markers. PCR primers (**Table 1**) were designed using Primer Premier 6.0 (GraphPad Software, San Diego, CA, USA) based on the sequence of each candidate of species markers.

**Table 1:** Species-specific primers designed in this study.

Primers	Primer sequence (5′-3′)	Target gene	Gene annotation	Start position^a^	Length (bp)
EC-F	TGAAAACCTTATCCGCGA	ECL_01098	Aminoacylase	15	397
EC-R	GGCAGGCTGGAAGATAAA			411	
EH-F	AACTGTCAGGGTTTGCGC	*pqqB* (LI64_09975)	Encoding redox active molecule	67	487
EH-R	CAAACAGCGCCACGTTAT			553	
ER-F	ATCAGCATCGGGATCGGT	EGY04_15785	Esterase family protein	34	1132
ER-R	GCTTTTGAACAAACTCAGCATA			1165	
EK-F	GGCATTGCCTTACAAGGAG	*gspE* (BFV64_21570)	Secretion ATPase	37	1403
EK-R	GTCACCCGCAGAATTTCT			1439	

a Base positions of the corresponding GenBank sequences at which primer sequences start.

### Establishment of Single and Multiplex PCR Systems

Species-specific primers were used to establish the single and multiplex PCR systems. Type strains (*E. cloacae* ssp. *cloacae* ATCC 13047, *E. hormaechei* ssp. *hormaechei* ATCC 49162, *E. roggenkampii* DSM 16690 and *E. kobei* DSM 13645) were used as positive controls. The mixture of a single PCR reaction (25 μl) contained 2×Taq Plus Master Mix II (Vazyme, Nanjing, China) (12.5 μl), 10 μM of each primer (1 μl), the DNA template (0.5 μl), and nuclease-free ultra-pure water (10 μl). The multiplex PCR mixture (50 μl) contained 25 μl of Vazyme 2×Taq Plus Master Mix II, 2 μl of primer mix containing 0.25 μl of each primer in 10 μM, 2 μl of DNA template, and 21 μl of nuclease-free ultra-pure water in a final volume of 50 μl. The reaction condition of single and multiplex PCR is identical, comprising an initial denaturation step at 95°C for 3 min, followed by 30 cycles of amplification (95°C for 15 s, 55°C for 20 s, and at 72°C for 90 s), and a final elongation step at 72°C for 5 min. PCR amplification was performed by using a Bio-Rad T100™ Thermal cycler (Bio-Rad, Hercules, USA). The PCR products were separated by using electrophoresis with 2.0% agarose gel for 40 min at 110 V in 1×Tris-Acetate-EDTA (TAE) buffer, and gels were visualized by using the gel imaging system instrument (Bio-Rad, Gel Doc™ XR^+^ with Image Lab™ Software). The total time cost of the PCR reactions and electrophoresis is approximately 150 min.

### Validation of the Specificity and Sensitivity of Established Single and Multiplex PCR Systems

231 strains of ECC were used to estimate the specificity and sensitivity of EC, EH, ER, and EK primers. To validate the specificity and sensitivity of multiplex PCR, 53 additional ECC isolates and 20 non-ECC isolates (four *Acinetobacter baumannii* strains, five *K. pneumoniae* strains, two *Serratia marcescens* strains, four *Escherichia coli* strains, two *Pseudomonas aeruginosa* strains, three *Flavobacterium meningosepticum* strains) recovered from different sources (63 isolated from clinical samples, eight isolated from hospital sewage water, and two isolated from feces of farmed animal) were tested. Type strains (*E. cloacae* ssp. *cloacae* ATCC 13047, *E. hormaechei* ssp. *hormaechei* ATCC 49162, *E. roggenkampii* DSM 16690 and *E. kobei* DSM 13645) were used as positive controls and *E. coli* ATCC25922 was used as the negative control.

### Species Identification

Species identification for the ECC members were performed by using *hsp60* typing as previously described (Hoffmann and Roggenkamp, 2003). Briefly, the fragments of *hsp60* gene of 284 ECC strains were amplified with using primers Hsp60*-*F (5′-GGTAGAAGAAGGCGTGGTTGC-3′) and Hsp60-R (5′-ATGCATTCGGTGGTGATCATCAG-3′) (Hoffmann and Roggenkamp, 2003) followed by Sanger sequencing. The 20 non-ECC isolated were identified by Vitek-II (bioMérieux) mass spectrometer and 16S rRNA gene sequencing. The results of ECC sequences were aligned and the phylogenetic tree was calculated by using MEGA X (Kumar et al., 2018). The *hsp60* sequences of 12 ECC clusters were included in the analysis as references, including cluster I-*E. asburiae* JCM 6051 (accession no. CP011863), cluster II-*E. kobei* ATCC BAA-260 (accession no. CP017181), cluster III-*E. hormaechei* subsp*. hoffmannii* DSM 14563 (accession no. CP017186), cluster IV-*E. roggenkampii* DSM 16690 (accession no. CP017184), cluster V-*E. ludwigii* EN-119 (accession no. CP017279), cluster VI-*E. hormaechei* subsp*. xiangfangensis* LMG 27195 (accession no. CP017183), cluster VII-*E. hormaechei* subsp*. hormaechei* ATCC 49162 (accession no. MKEQ00000000), cluster VIII-*E. hormaechei* subsp*. steigerwaltii* DSM 16691 (accession no. CP017179), cluster IX-*E. bugandensis* EB-247 (accession no. FYBI00000000), cluster XI-*E. cloacae* subsp*. cloacae* ATCC 13047 (accession no. CP001918) and cluster XII-*E. cloacae* subsp*. dissolvens* ATCC 23373 (accession no. WJWQ00000000).

The untypeable strains by *hsp60* typing method were further sent to WGS to confirm the species. Genomic DNA was extracted using a Gentra Puregene Yeat/Bact. Kit (Qiagen, San Francisco/Bay area, CA, USA), and subjected to WGS by using Illumina novaseq 6000 system (Illumina, San Diego, United States), using 2×150-bp pair-end libraries. Illumina reads were trimmed using Trimmomatic (Bolger et al., 2014) (REF or Website) and assembled with SPAdes v3.12.0 (Bankevich et al., 2012). ParSNP v1.2 (Treangen et al., 2014) was used to align the core genome to calculate the phylogenetic tree with settings: min LCB size 25, maximal diagonal difference 12% and enabling filtering of SNPs located in PhiPack identified regions of recombination.

### Evaluation of the Multiplex PCR Applicability for DNA Amount

Type strains of four species were used to estimate the detection limit of the multiplex PCR established here. The analytical sensitivity of the multiplex PCR using DNA directly extracted from tested strains which serially diluted ranged from 1000 to 1 pg. After amplification, 5 μl of each PCR product was loaded onto 2.0% agarose gels. The detection limit was determined to be the smallest DNA quantity that generated the correct band pattern for the targeted gene.

### Statistical Analyses

Specificity and sensitivity were used to quantify the identification accuracy of species-specific primers designed in this study. The specificity is the proportion of true negatives that are correctly identified by the primer tests, and sensitivity is the proportion of true positives that are correctly identified by the primer tests. The results of species-specific PCRs were compared with that of *hsp60* typing. Specificity and sensitivity were measured with a 95% confidence interval (95% CI) based on true positives (TP), true negatives (TN), false positives (FP), and false negatives (FN) (Lalkhen and McCluskey, 2008). The specificity as TN/(TN + FP), while sensitivity was calculated as TP/(TP + FN). The sensitivity and specificity values were calculated using MedCalc Software^1^.

## Results

### Design of Species-Specific Primers for *E. cloacae, E. hormaechei, E. roggenkampii, and E. kobei*

A collection of 977 ECC genomes were retrieved from the NCBI RefSeq database. According to the ANI results, 808 genomes were assigned to *E. hormaechei* (n=714), *E. cloacae* (n=50), *E. kobei* (n=26), and *E. roggenkampii* (n=18). Pan-genome construction of each species was conducted to query the species-specific core genes. To avoid high sequence homology between all of the candidate unique genes which might be a consequence of the high identity threshold chosen for pan-genome construction, and to obtain species-specific primers with high possibility, the pair-wise blast was further performed. Four species-specific genes were obtained for the four species, respectively, including a gene (ECL_01098) encoding an aminoacylase (Curley and van Sinderen, 2000; Dettori et al., 2018) in the genome of *E. cloacae* ssp. *cloacae* strain ATCC 13047 (accession no. CP001918.1); the *pqqB* (LI64_09975) gene encoding a small redox active molecule (Shen et al., 2012) in the genome of *E. hormaechei* ssp. *hormaechei* strain 34983 (accession no. CP010377.1); a gene (EGY04_15785) encoding an esterase family protein (Bornscheuer, 2002) in the genome of *E. roggenkampii* strain FDAARGOS_523 (accession no. CP033800.1); and the secretion ATPase gene *gspE* (BFV64_21570) (Lu et al., 2013) in the genome of *E. kobei* strain DSM 13645 (accession no. CP017181.1).

**Figure 1:**
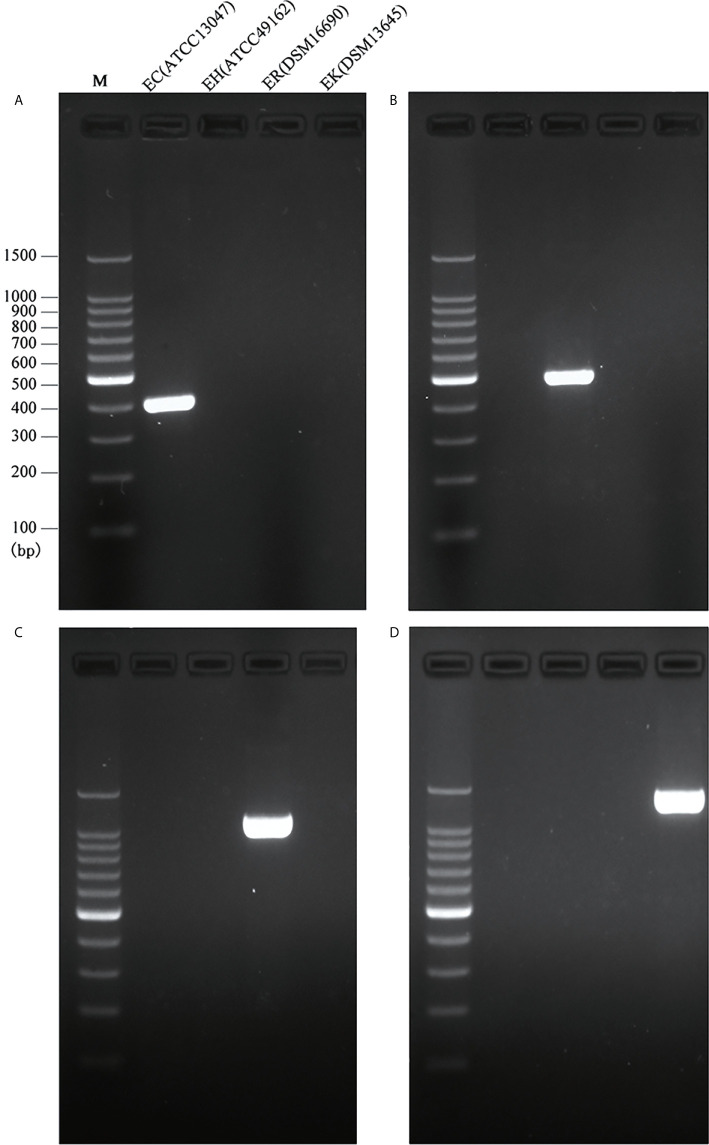
Species-specific PCRs for the identification of *E. cloacae*, *E*. *hormaechei E. roggenkampii* and *E. kobei*. **(A)** Products of single PCRs with primers EC-F/R and the four type strains; **(B)** Products of single PCRs with primers EH-F/R and the four type strains; **(C)** Products of single PCRs with primers ER-F/R and the four type strains; **(D)** Products of single PCRs with primers EK-F/R and the four type strains.

The full length of each species-unique gene was extracted from genomes of each species and aligned to obtain the conserved fragments for the design of species-specific primers. This resulted in four pairs of primers as listed in **Table 1**. *In silico* PCR showed that the primers generated products with different sizes, i.e. 397 bp for *E. cloacae*, 487 bp for *E. hormaechei*, 1132 bp for *E. roggenkampii*, and 1403 bp for *E. kobei* (**Table 1** and **Figure 1**).

### Evaluation of the Specificity and Sensitivity for Designed Species-Specific Primers

A total of 231 ECC strains were initially typed by using *hsp60* typing. All of the strains were tested by using the primers EC-F/R, EH-F/R, ER-F/R, and EK-F/R. PCR results showed that 12 strains were positive and 219 were negative to the primers EC-F/R, which were fully consistent with that of *hsp60* typing (specificity: 100.00%; sensitivity: 100.00%). PCR results of the primers EH-F/R showed that 158 were positive and 73 were negative. Compared with the results of *hsp60* typing, one positive strain was classified as false positive (specificity: 98.51%; sensitivity: 95.73%). The tests by using the primers ER-F/R identified 16 positive samples and 215 negative samples. Three false positives and four false negatives were identified by *hsp60* typing (specificity: 98.60%; sensitivity: 76.47%). Lastly, 31 were positive and 200 were negative to the primers EK-F/R. The *hsp60* typing results showed that seven were false positives and two were false negatives (specificity, 96.59%; sensitivity, 92.31%). The results were detailed in **Table 2** and **Table S1**.

**Table 2:** Specificity and sensitivity of species-specific primers designed in this study.

Primers	Test result					Specificity (100%)		Sensitivity (100%)	
	True Positive	False Positive	True Negative	False Negative	Total	Value	95% CI	Value	95% CI
EC-F/R	12	0	219	0	231	100.00	98.33–100.00	100.00	73.54–100.00
EH-F/R	157	1	66	7	231	98.51	91.96–99.96	95.73	91.40–98.27
ER-F/R	13	3	211	4	231	98.60	95.96–99.71	76.47	50.10–93.19
EK-F/R	24	7	198	2	231	96.59	93.09–98.62	92.31	74.87–99.05
Multiplex	43	0	30	0	73	100.00	88.43–100.00	100.00	91.78–100.00

### Development and Validation of Multiplex PCR Assay

**Figure 2:**
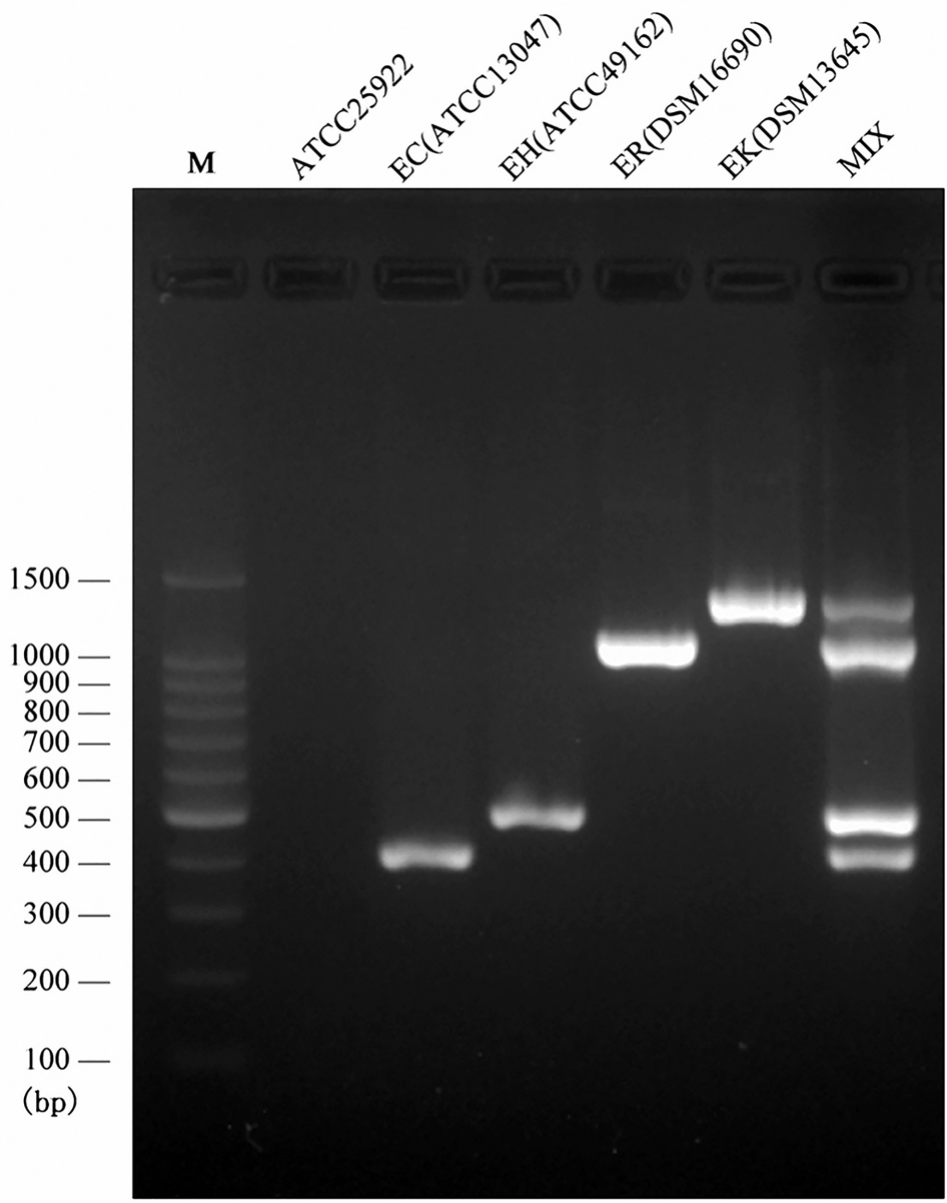
Multiplex PCR assay for the identification of four species belonging to ECC. Visualization of the PCR products on an agarose (2.0%) gel for four type strains. Lane M, DNA ladder; Lan1, negative control; Lane 2, the PCR product derived from primers EC-F/R and *E. cloacae* ssp. *cloacae* ATCC 13047; lane 3, the PCR product derived from primers EH-F/R and *E. hormaechei* ssp. *hormaechei* ATCC 49162; lane 4, the PCR product derived from primers ER-F/R and *E. roggenkampii* DSM 16690; lane 5, the PCR product derived from primers EK-F/R and *E. kobei* DSM 13645; Lane 6, the multiplex PCR product.

Following the validated single species-specific PCRs, a multiplex PCR system using the four pairs of primers was set up. The product of four type strains using the multiplex PCR system was separated well by the electrophoresis with 2.0% agarose gel, resulting in four bands (**Figure 2**). We further evaluated the multiplex PCR system by testing 53 ECC strains which were isolated from clinical samples, sewage water, and animal feces and 20 non-ECC strains (four *Acinetobacter baumannii*, five *K. pneumoniae*, two *Serratia marcescens*, four *Escherichia coli*, two *Pseudomonas aeruginosa*, three *Flavobacterium meningosepticum*). The established multiplex PCR identified 1 isolate as *E. cloacae*, 17 as *E. hormaechei*, 16 as *E. roggenkampii*, 9 as *E. kobei*, and the remaining 10 ECC strains and 20 non-ECC strains were negative to the PCR reactions (**Table 3**). The *hsp60* sequences of six ECC strains (1339, 1340, 1342, 1383, 6069, and 12740) clustered together without any of type strains, thus failed being typed. We further performed WGS for two of them (1339 and 1383) to identify the species, and the phylogenetic analysis showed that they belonged to *E. asburiae* (data not shown). Collectively, the results of the multiplex PCR are fully consistent with those of *hsp60* typing and WGS (**Table 3**). The multiplex PCR assay showed 100% sensitivity (95% CI 91.78–100%) and 100% specificity (95% CI 88.43–100%) for the identification of *E. cloacae*, *E. hormaechei*, *E. roggenkampii*, and *E. kobei* (**Table 2**).

**Table 3:** Results of the multiplex PCR for the detection of 73 strains.

Strain ID	Source	Results by *hsp60* typing or MS	Results by the multiplex PCR
1	Clinical sample	*A. baumannii*	N^a^
2	Clinical sample	*E. hormaechei*	EH
3	Clinical sample	*A. baumannii*	N
4	Clinical sample	*K. pneumoniae*	N
5	Clinical sample	*K. pneumoniae*	N
6-3	Sewage water	*E. roggenkampii*	ER
5-6	Sewage water	*E. roggenkampii*	ER
8-4	Sewage water	*E. asburiae*	N
86	Clinical sample	*A. baumannii*	N
87	Clinical sample	*A. baumannii*	N
93	Clinical sample	*S. marcescens*	N
113	Clinical sample	*E. coli*	N
119	Clinical sample	*S. marcescens*	N
120	Clinical sample	*E. coli*	N
262	Clinical sample	*E. coli*	N
289	Clinical sample	*P. aeruginosa*	N
422	Sewage water	*E. kobei*	EK
430	Clinical sample	*F. meningosepticum*	N
435	Clinical sample	*F. meningosepticum*	N
436	Clinical sample	*F. meningosepticum*	N
437	Clinical sample	*K. pneumoniae*	N
438	Clinical sample	*K. pneumoniae*	N
439	Clinical sample	*K. pneumoniae*	N
689	Animal feces	*E. hormaechei*	EH
690	Animal feces	*E. hormaechei*	EH
695	Clinical sample	*P. aeruginosa*	N
981	Sewage water	*E. roggenkampii*	ER
982	Sewage water	*E. roggenkampii*	ER
983	Sewage water	*E. roggenkampii*	ER
1122	Clinical sample	*E. hormaechei*	EH
1123	Clinical sample	*E. hormaechei*	EH
1135	Clinical sample	*E. roggenkampii*	ER
1136	Clinical sample	*E. roggenkampii*	ER
1140	Clinical sample	*E. kobei*	EK
1146	Clinical sample	*E. roggenkampii*	ER
1166	Clinical sample	*E. asburiae*	N
1167	Clinical sample	*E. coli*	N
1187	Sewage water	*E. roggenkampii*	ER
1276	Clinical sample	*E. kobei*	EK
1277	Clinical sample	*E. kobei*	EK
1278	Clinical sample	*E. kobei*	EK
1279	Clinical sample	*E. roggenkampii*	ER
1280	Clinical sample	*E. roggenkampii*	ER
1281	Clinical sample	*E. roggenkampii*	ER
1282	Clinical sample	*E. kobei*	EK
1335	Clinical sample	*E. roggenkampii*	ER
1336	Clinical sample	*E. asburiae*	N
1337	Clinical sample	*E. kobei*	EK
1338	Clinical sample	*E. roggenkampii*	ER
1339^b,c^	Clinical sample	*E. asburiae*	N
1340^c^	Clinical sample	*E. asburiae*	N
1341	Clinical sample	*E. kobei*	EK
1342^c^	Clinical sample	*E. asburiae*	N
1372	Clinical sample	*E. hormaechei*	EH
1377	Clinical sample	*E. hormaechei*	EH
1378	Clinical sample	*E. hormaechei*	EH
1379	Clinical sample	*E. roggenkampii*	ER
1380	Clinical sample	*E. roggenkampii*	ER
1381	Clinical sample	*E. hormaechei*	EH
1382	Clinical sample	*E. hormaechei*	EH
1383^b,c^	Clinical sample	*E. asburiae*	N
1384	Clinical sample	*E. cloacae*	EC
1385	Clinical sample	*E. hormaechei*	EH
1386	Clinical sample	*E. hormaechei*	EH
1387	Clinical sample	*E. hormaechei*	EH
1388	Clinical sample	*E. hormaechei*	EH
1389	Clinical sample	*E. hormaechei*	EH
1390	Clinical sample	*E. hormaechei*	EH
8467	Clinical sample	*E. kobei*	EK
12740^c^	Clinical sample	*E. asburiae*	N
6069^c^	Clinical sample	*E. asburiae*	N
6530	Clinical sample	*E. hormaechei*	EH
9636	Clinical sample	*E. asburiae*	N

a N, Negative.

b Strains were sequenced by WGS.

c The hsp60 sequences of strains clustered together without any of type sequences.

### Evaluation of the Detection Limit for Multiplex PCR

**Figure 3:**
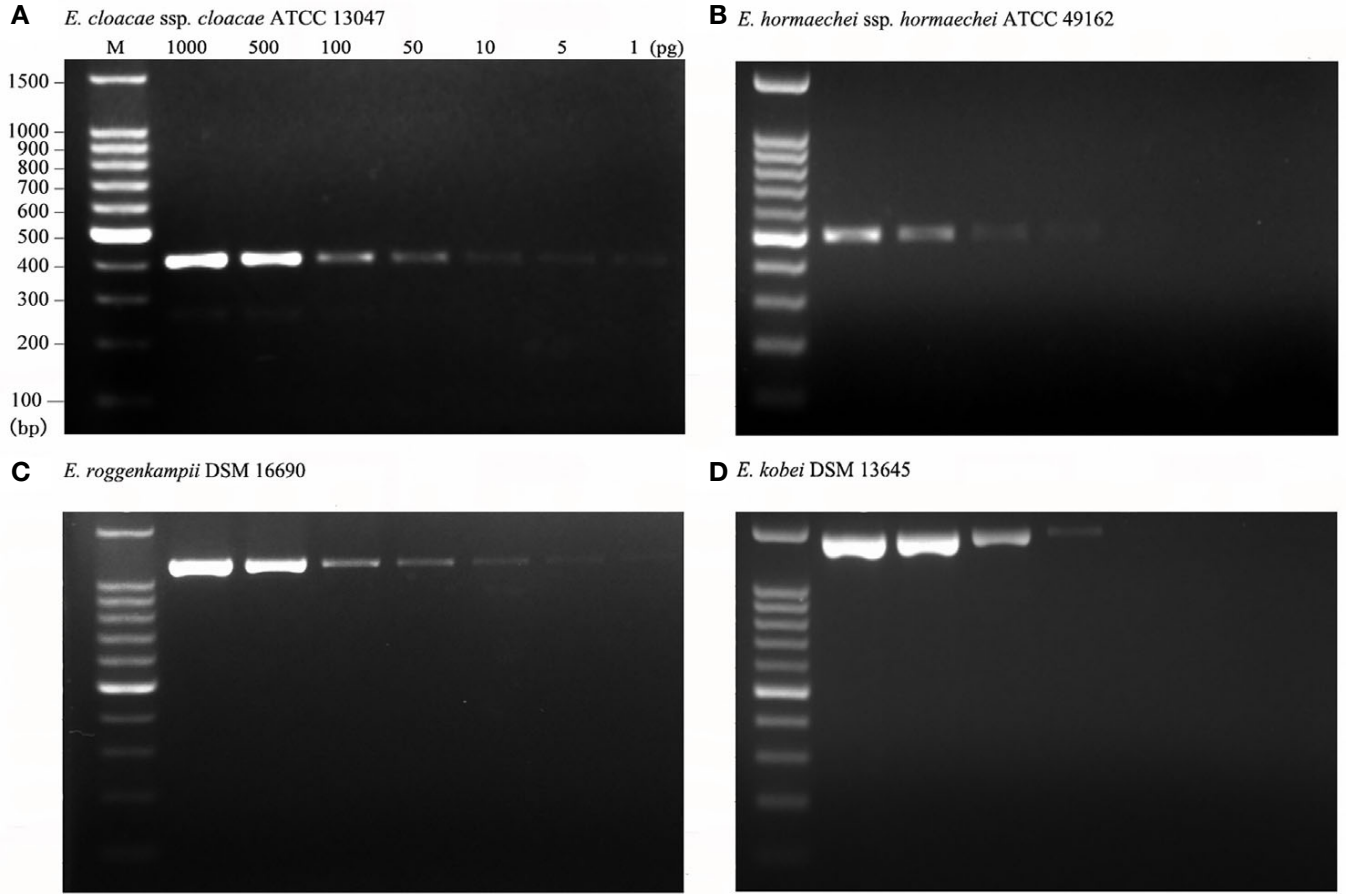
Analytical sensitivity of the multiplex PCR. Analytical sensitivity was evaluated for the type strains of four species using 1 to 1,000pg DNA per reaction mixture. The analytical sensitivities were 1 pg for *E. cloacae* ssp. *cloacae* ATCC 13047 **(A)**; 50 pg for *E. hormaechei* ssp. *hormaechei* ATCC 49162 **(B)**; 5 pg for *E. roggenkampii* DSM 16690 **(C)**; 50 pg for *E. kobei* DSM 13645 **(D)**.

We further examined the detection limit for the multiplex PCR employed in the clinical setting by using serially diluted DNA of isolates of the targeted ECC. The detectable DNA amount was 1 pg for *E. cloacae* ssp. *cloacae* ATCC 13047 (**Figure 3A**), 50 pg for *E. hormaechei* ssp. *hormaechei* ATCC 49162 (**Figure 3B**), 5 pg for *E. roggenkampii* DSM 16690 (**Figure 3C**) and 50 pg for *E. kobei* DSM 13645 (**Figure 3D**). The data prove that the multiplex PCR established in this study is with very high analytical sensitivity.

## Discussion

According to the ANI results of 977 ECC genomes retrieved from the NCBI RefSeq database, the four species (*E. cloacae*, *E. hormaechei*, *E. roggenkampii*, and *E. kobei*) accounted for 82.7% of ECC population studied in this work, and most genomes were clinically associated, suggesting that the four species were predominant in the clinical setting. This is consistent with the previous studies (Zhou et al., 2017; Davin-Regli et al., 2019; Wang et al., 2020). It is meaningful to develop a method which can identify ECC organisms efficiently and differentially, and has the potential to aid the control of four major ECC infections greatly. In the present study, we tried to develop a simple and effective multiplex PCR assay that enables the differential identification of the four ECC species. The species-specific core genes of the four ECC species detected by comparative genomics provide meaningful markers for their identification. All primers were newly designed and optimized for development of the multiplex PCR assay in this study.

In clinical laboratories, various methods for identifying ECC species have been introduced over the past decades, and molecular techniques and MALDI-TOF MS are frequently used to identify ECC species (Hoffmann and Roggenkamp, 2003; Paauw et al., 2008; Ohad et al., 2014; Chavda et al., 2016; Singh et al., 2018; Davin-Regli et al., 2019). These techniques reduce the error rates for the results, thereby enabling the acceleration of treatment decisions according to the accurate identifications. MALDI-TOF MS, microarray-CGH, MLSA, WGS, and qPCR technologies have been surpassed by *hsp60* typing as the methods of choice for rapidly discriminating the species of this genus. The one-step multiplex PCR platform is a kind of common PCR, and it does not require additional steps. Researchers had developed one-step multiplex PCR methods to solve the clinical problems for the accurate identification of mycobacteria at the species and/or subspecies level, and it has demonstrated 100% specificity for the targeted Mycobacterium species (Chae et al., 2017). Similar as what we did here, a multiplex PCR method has been developed to identify three species (*K. pneumoniae*, *K. variicola*, and *K. quasipneumoniae*) of *Klebsiella* genus, and has been verified that it is feasible to carry out *Klebsiella* identification in the clinical routine work (Fonseca et al., 2017). Although several multiplex PCR systems of some species have already been commercialized and introduced into laboratories to do strain classifications (Brito et al., 2003; Chae et al., 2017; Fonseca et al., 2017), to our knowledge, there were no multiplex PCR assays capable of simultaneously discriminating various ECC species with clinically importance.^1^ 1
https://www.medcalc.org/calc/diagnostic_test.php



We initially performed single PCRs to evaluate the specificity and sensitivity for each pair of species-specific primers designed in this study. A unique band with expected size presented in each single PCR detection by using primers EC-F/R for *E. cloacae* ATCC 13047 (**Figure 1A**), EH-F/R for *E. hormaechei* ATCC 49162 (**Figure 1B**), ER-F/R for *E. roggenkampii* DSM 16690 (**Figure 1C**), and EK-F/R for *E. kobei* DSM 13645 (**Figure 1D**). A large set of clinical strains were further tested for the primers to estimate their specificity and sensitivity. Our tests showed that the specificity and sensitivity of our primers were reliable with a 95% confidence interval. The sensitivity of ER-F/R and EK-F/R is lower than that of EC-F/R and EH-F/R. We suppose that the reason could be a limited number of genomes available in the 1
https://www.medcalc.org/calc/diagnostic_test.phpChinapublic database for the two species (26 genomes of *E. kobei*, and 17 of *E. roggenkampii*), resulting in a relatively low sensitivity. As the accumulation of genomes in the public database, the primers ER-F/R and EK-F/R could be updated in the near future. Of note, the multiplex PCR designed in our study currently is culture dependent. To accelerate microbial identification, direct detection from clinical specimens is a promising strategy. Therefore, it is worth evaluating the capacity of our multiplex PCR for culture-independent detections in the future.

In summary, we here developed and validated a novel multiplex PCR amplification method that can simultaneously identify four major members of ECC (i.e. *E. cloacae*, *E. hormaechei*, *E. roggenkampii*, and *E. kobei*) with clinical significance. In comparison to available molecular methods, our method is rapid, accurate, and cost-efficient which is suitable for being used in routine diagnostic tests. Additionally, the application of the established multiplex PCR method would highly facilitate us to understand the ECC in the aspect of clinical significance, epidemiology, and drug resistance at the species level.

## Data Availability Statement

The data sets presented in this study can be found in online repositories. The names of the repository/repositories and accession number(s) can be found below: https://www.ncbi.nlm.nih.gov/, JAEKKA000000000 https://www.ncbi.nlm.nih.gov/, JAEKKB000000000.

## Author Contributions

YJ: strain collection, run all tests, and writing—original draft. PW: strain genome data collection and comparison, primer design, and genome data analysis. TX: experimental design, writing—review and editing, and statistical analysis. YZ: project administration. RC: statistical analysis. HZ and KZ: conceptualization, writing—review and editing, project administration, resources, and supervision. All authors contributed to the article and approved the submitted version.

## Funding

This work was supported by the National Key Research and Development Program of China (2017YFC1200200), Major Infectious Diseases Such as AIDS and Viral Hepatitis Prevention and Control Technology Major Projects (2018ZX10712-001), the National Natural Science Foundation of China (81702045 and 81902030), and Shenzhen Basic Research projects (JCYJ20190807144409307).

## Conflict of Interest

Authors YJ, PW, TX, YZ, HZ, KZ have a pending application of the patent for the multiplex PCR.

The remaining authors declare that the research was conducted in the absence of any commercial or financial relationships that could be construed as a potential conflict of interest.
