# Supplementary material for: Development of a One-Step Multiplex PCR Assay for Differential Detection of Four species (Enterobacter cloacae, Enterobacter hormaechei, Enterobacter roggenkampii, and Enterobacter kobei) Belonging to Enterobacter cloacae Complex With Clinical Significance
Source: Front Cell Infect Microbiol. 2021 May 18;11:677089. doi: 10.3389/fcimb.2021.677089 (PMC8169972; doi:10.3389/fcimb.2021.677089)
Supplement: Supplementary file 1 [file Table_1.docx]

**Table S1** **Results of *hsp60* typing and species-specific PCRs for the detection of 231 ECC strains**

| **Strain ID** | ***hsp60* typing** | **EC-F/R**  **primers** | **EH-F/R primers** | **ER-F/R primers** | **EK-F/R primers** |
| --- | --- | --- | --- | --- | --- |
| 6 | *E. kobei* | N^a^ | N | N | N |
| 7 | *E. hormaechei* | N | P^b^ | N | N |
| 8 | *E. hormaechei* | N | P | N | N |
| 9 | *E. cancerogenus* | N | N | N | N |
| 10 | *E. hormaechei* | N | P | N | N |
| 11 | *E. kobei* | N | N | P | P |
| 12 | *E. hormaechei* | N | P | N | N |
| 13 | *E. asburiae* | N | N | N | N |
| 14 | *E. ludwigii* | N | N | N | N |
| 15 | *E. cloacae* | P | N | N | N |
| 16 | *E. hormaechei* | N | P | N | N |
| 17 | *E. hormaechei* | N | P | N | N |
| 18 | *E. hormaechei* | N | P | N | N |
| 18 | *E. hormaechei* | N | P | N | N |
| 19 | *E. hormaechei* | N | P | N | N |
| 20 | *E. hormaechei* | N | P | N | N |
| 21 | *E. hormaechei* | N | P | N | N |
| 22 | *E. hormaechei* | N | P | N | N |
| 23 | *E. bugandensis* | N | N | N | N |
| 24 | *E. kobei* | N | N | N | P |
| 25 | *E. hormaechei* | N | P | N | N |
| 26 | *E. hormaechei* | N | P | N | N |
| 27 | *E. hormaechei* | N | P | N | N |
| 28 | *E. hormaechei* | N | P | N | N |
| 29 | *E. hormaechei* | N | P | N | N |
| 30 | *E. hormaechei* | N | P | N | N |
| 31 | *E. hormaechei* | N | P | N | N |
| 32 | *E. hormaechei* | N | P | N | N |
| 33 | *E. hormaechei* | N | P | N | N |
| 34 | *E. hormaechei* | N | P | N | N |
| 35 | *E. hormaechei* | N | P | N | N |
| 36 | *E. asburiae* | N | N | N | N |
| 37 | *E. hormaechei* | N | P | N | N |
| 38 | *E. hormaechei* | N | P | N | N |
| 39 | *E. hormaechei* | N | P | N | N |
| 40 | *E. hormaechei* | N | P | N | N |
| 41 | *E. hormaechei* | N | P | N | N |
| 42 | *E. hormaechei* | N | P | N | N |
| 43 | *E. hormaechei* | N | P | N | N |
| 44 | *E. hormaechei* | N | P | N | N |
| 45 | *E. hormaechei* | N | P | N | N |
| 46 | *E. hormaechei* | N | P | N | N |
| 47 | *E. hormaechei* | N | P | N | N |
| 48 | *E. hormaechei* | N | P | N | N |
| 49 | *E. hormaechei* | N | P | N | N |
| 50 | *E. hormaechei* | N | P | N | N |
| 51 | *E. hormaechei* | N | P | N | N |
| 52 | *E. hormaechei* | N | P | N | N |
| 53 | *E. hormaechei* | N | P | N | N |
| 54 | *E. hormaechei* | N | P | N | N |
| 55 | *E. hormaechei* | N | P | N | N |
| 56 | *E. hormaechei* | N | P | N | N |
| 57 | *E. roggenkampii* | N | N | N | N |
| 58 | *E. hormaechei* | N | P | N | N |
| 59 | *E. hormaechei* | N | P | N | N |
| 60 | *E. hormaechei* | N | P | N | N |
| 61 | *E. hormaechei* | N | P | N | P |
| 62 | *E. hormaechei* | N | P | N | N |
| 63 | *E. hormaechei* | N | P | N | N |
| 64 | *E. hormaechei* | N | P | N | N |
| 65 | *E. hormaechei* | N | P | N | N |
| 66 | *E. hormaechei* | N | P | N | N |
| 67 | *E. hormaechei* | N | P | N | N |
| 68 | *E. hormaechei* | N | P | N | N |
| 69 | *E. hormaechei* | N | P | N | N |
| 70 | *E. hormaechei* | N | P | N | N |
| 71 | *E. hormaechei* | N | P | N | N |
| 72 | *E. kobei* | N | N | N | P |
| 73 | *E. hormaechei* | N | P | N | N |
| 116 | *E. hormaechei* | N | P | N | N |
| 117 | *E. hormaechei* | N | P | N | N |
| 121 | *E. cloacae* | P | N | P | N |
| 122 | *E. hormaechei* | N | P | N | N |
| 123 | *E. kobei* | N | N | N | P |
| 124 | *E. hormaechei* | N | P | N | N |
| 125 | *E. kobei* | N | N | N | P |
| 126 | *E. hormaechei* | N | P | N | N |
| 127 | *E. roggenkampii* | N | N | P | N |
| 128 | *E. hormaechei* | N | P | N | N |
| 129 | *E. hormaechei* | N | P | N | N |
| 130 | *E. hormaechei* | N | P | N | N |
| 131 | *E. hormaechei* | N | P | N | N |
| 132 | *E. hormaechei* | N | P | N | N |
| 133 | *E. hormaechei* | N | P | N | N |
| 134 | *E. hormaechei* | N | P | N | N |
| 135 | *E. hormaechei* | N | P | N | N |
| 136 | *E. hormaechei* | N | P | N | N |
| 137 | *E. kobei* | N | N | N | P |
| 138 | *E. kobei* | N | N | N | P |
| 139 | *E. hormaechei* | N | P | N | N |
| 140 | *E. roggenkampii* | N | P | P | N |
| 141 | *E. hormaechei* | N | P | N | N |
| 142 | *E. hormaechei* | N | P | N | N |
| 143 | *E. asburiae* | N | N | N | N |
| 144 | *E. kobei* | N | N | N | P |
| 145 | *E. kobei* | N | N | N | P |
| 146 | *E. kobei* | N | N | N | P |
| 147 | *E. hormaechei* | N | P | N | P |
| 148 | *E. hormaechei* | N | P | N | P |
| 149 | *E. hormaechei* | N | P | N | P |
| 150 | *E. hormaechei* | N | P | N | N |
| 151 | *E. hormaechei* | N | P | N | P |
| 152 | *E. hormaechei* | N | P | N | P |
| 153 | *E. hormaechei* | N | P | N | N |
| 154 | *E. hormaechei* | N | P | N | N |
| 155 | *E. hormaechei* | N | P | N | N |
| 156 | *E. hormaechei* | N | P | N | N |
| 157 | *E. kobei* | N | N | N | P |
| 158 | *E. hormaechei* | N | P | N | N |
| 159 | *E. hormaechei* | N | P | N | P |
| 160 | *E. kobei* | N | N | N | P |
| 161 | *E. hormaechei* | N | P | N | N |
| 162 | *E. hormaechei* | N | P | N | N |
| 163 | *E. hormaechei* | N | P | N | N |
| 164 | *E. roggenkampii* | N | N | P | N |
| 165 | *E. cloacae* | P | N | N | N |
| 166 | *E. kobei* | N | N | N | P |
| 167 | *E. hormaechei* | N | P | N | N |
| 168 | *E. hormaechei* | N | P | N | N |
| 169 | *E. hormaechei* | N | P | N | N |
| 170 | *E. hormaechei* | N | P | N | N |
| 171 | *E. hormaechei* | N | P | N | N |
| 172 | *E. hormaechei* | N | P | N | N |
| 173 | *E. hormaechei* | N | P | N | N |
| 174 | *E. hormaechei* | N | P | N | N |
| 175 | *E. hormaechei* | N | P | N | N |
| 176 | *E. hormaechei* | N | P | N | N |
| 177 | *E. hormaechei* | N | P | N | N |
| 178 | *E. hormaechei* | N | P | N | N |
| 179 | *E. hormaechei* | N | P | N | N |
| 180 | *E. hormaechei* | N | P | N | N |
| 181 | *E. roggenkampii* | N | N | P | N |
| 182 | *E. hormaechei* | N | P | N | N |
| 184 | *E. hormaechei* | N | P | N | N |
| 185 | *E. hormaechei* | N | P | N | N |
| 186 | *E. hormaechei* | N | P | N | N |
| 187 | *E. hormaechei* | N | P | N | N |
| 188 | *E. hormaechei* | N | P | N | N |
| 189 | *E. hormaechei* | N | P | N | N |
| 190 | *E. cloacae* | P | N | N | N |
| 191 | *E. cloacae* | P | N | N | N |
| 192 | *E. hormaechei* | N | P | N | N |
| 193 | *E. hormaechei* | N | P | N | N |
| 194 | *E. hormaechei* | N | P | N | N |
| 195 | *E. ludwigii* | N | N | N | N |
| 196 | *E. hormaechei* | N | P | N | N |
| 197 | *E. hormaechei* | N | P | N | N |
| 198 | *E. roggenkampii* | N | N | P | N |
| 199 | *E. cloacae* | P | N | N | N |
| 200 | *E. cloacae* | P | N | N | N |
| 201 | *E. roggenkampii* | N | N | P | N |
| 202 | *E. hormaechei* | N | P | N | N |
| 203 | *E. hormaechei* | N | P | N | N |
| 204 | *E. hormaechei* | N | N | N | N |
| 205 | *E. hormaechei* | N | P | N | N |
| 206 | *E. hormaechei* | N | P | N | N |
| 207 | *E. kobei* | N | N | N | N |
| 208 | *E. cloacae* | P | N | N | N |
| 209 | *E. hormaechei* | N | P | N | N |
| 210 | *E. hormaechei* | N | P | P | N |
| 211 | *E. hormaechei* | N | P | N | N |
| 212 | *E. hormaechei* | N | P | N | N |
| 213 | *E. kobei* | N | N | N | P |
| 214 | *E. hormaechei* | N | P | N | N |
| 215 | *E. hormaechei* | N | P | N | N |
| 216 | *E. hormaechei* | N | N | N | N |
| 217 | *E. kobei* | N | N | N | P |
| 218 | *E. hormaechei* | N | P | N | N |
| 219 | *E. hormaechei* | N | P | N | N |
| 220 | *E. roggenkampii* | N | N | P | N |
| 221 | *E. kobei* | N | N | N | P |
| 222 | *E. kobei* | N | N | N | P |
| 223 | *E. kobei* | N | N | N | P |
| 224 | *E. hormaechei* | N | P | N | N |
| 225 | *E. hormaechei* | N | P | N | N |
| 226 | *E. hormaechei* | N | P | N | N |
| 227 | *E. hormaechei* | N | P | N | N |
| 228 | *E. hormaechei* | N | P | N | N |
| 229 | *E. hormaechei* | N | P | N | N |
| 230 | *E. hormaechei* | N | P | N | N |
| 231 | *E. cloacae* | N | N | N | N |
| 232 | *E. roggenkampii* | N | N | P | N |
| 233 | *E. hormaechei* | N | N | N | N |
| 234 | *E. cloacae* | P | N | N | N |
| 235 | *E. hormaechei* | N | P | N | N |
| 236 | *E. hormaechei* | N | N | N | N |
| 237 | *E. hormaechei* | N | N | N | N |
| 238 | *E. hormaechei* | N | P | N | N |
| 239 | *E. hormaechei* | N | P | N | N |
| 240 | *E. hormaechei* | N | P | N | N |
| 241 | *E. hormaechei* | N | P | N | N |
| 242 | *E. hormaechei* | N | P | N | N |
| 243 | *E. kobei* | N | N | N | P |
| 244 | *E. hormaechei* | N | P | N | N |
| 245 | *E. hormaechei* | N | P | N | N |
| 246 | *E. roggenkampii* | N | N | N | N |
| 247 | *E. bugandensis* | N | N | N | N |
| 248 | *E. hormaechei* | N | P | N | N |
| 249 | *E. hormaechei* | N | P | N | N |
| 250 | *E. cloacae* | P | N | N | N |
| 251 | *E. roggenkampii* | N | N | P | N |
| 252 | *E. hormaechei* | N | P | N | N |
| 253 | *E. hormaechei* | N | P | N | N |
| 254 | *E. kobei* | N | N | N | P |
| 255 | *E. hormaechei* | N | P | N | N |
| 256 | *E. hormaechei* | N | P | N | N |
| 257 | *E. hormaechei* | N | P | N | N |
| 258 | *E. hormaechei* | N | P | N | N |
| 259 | *E. ludwigii* | N | N | N | N |
| 260 | *E. hormaechei* | N | P | N | N |
| 261 | *E. hormaechei* | N | P | N | N |
| 264 | *E. hormaechei* | N | P | N | N |
| 265 | *E. hormaechei* | N | N | N | N |
| 266 | *E. kobei* | N | N | N | P |
| 267 | *E. roggenkampii* | N | N | N | N |
| 268 | *E. roggenkampii* | N | N | N | N |
| 269 | *E. kobei* | N | N | N | P |
| 270 | *E. kobei* | N | N | N | P |
| 271 | *E. roggenkampii* | N | N | P | N |
| 272 | *E. roggenkampii* | N | N | P | N |
| 273 | *E. hormaechei* | N | N | N | N |
| 274 | *E.cloacae* | P | N | N | N |
| 275 | *E. roggenkampii* | N | N | P | N |
| 276 | *E. kobei* | N | N | N | P |
| 277 | *E. hormaechei* | N | N | N | N |
| 278 | *E. cloacae* | P | N | N | N |
| 279 | *E. hormaechei* | N | P | N | N |
| 282 | *E. cloacae* | N | N | N | N |
| 285 | *E. roggenkampii* | N | N | P | N |
| 286 | *E. hormaechei* | N | P | N | N |
| 287 | *E. hormaechei* | N | P | N | N |

^a^N: Negative

^b^P: Positive
